# Role of the Bone Marrow Niche in Supporting the Pathogenesis of Lymphoid Malignancies

**DOI:** 10.3389/fcell.2021.692320

**Published:** 2021-07-28

**Authors:** Shahrzad Jalali, Stephen M. Ansell

**Affiliations:** Division of Hematology and Internal Medicine, Mayo Clinic, Rochester, MN, United States

**Keywords:** bone marrow niche, Waldenstrom Macroglobulinemia, follicular lymphoma, marginal zone lymphoma, mantle cell lymphoma, diffuse large B-cell lymphoma

## Abstract

While the bone marrow (BM) microenvironment is the primary location for nurturing the multipotent hematopoietic stem cells and developing the blood cells of either myeloid or lymphoid origin under normal physiological conditions, it could provide a supportive milieu for the proliferation of blood cancer cells. In fact, the multiple and complex direct cell-to-cell or indirect soluble factors-mediated interactions taking place among the BM cells of different origins are shown to play a significant role in tumorigenesis of hematological cancers. In the current review, we focus on lymphoid malignancies and highlight the novel insights surrounding the role of both cellular as well as non-cellular BM compartments in modulating hematopoiesis and promoting growth and proliferation of cancer cells across a variety of aggressive and indolent lymphoid malignancies, including diffuse large B-cell lymphoma, follicular lymphoma, mantle cell lymphoma, and Waldenstrom Macroglobulinemia. We also discuss the mechanisms of potential intervention and discuss their therapeutic impact in clinical settings.

## Introduction

Described as the primary location for hematopoiesis, the bone marrow (BM) niche is conventionally divided into two major cellular compartments, comprising of hematopoietic cells at different stages of differentiation and the stromal component, which is composed of mesenchymal stromal cells (MSCs) and heterogeneous cell populations of bone cells, neural cells, fibroblasts, adipocytes, and endothelial cells. Early evidences have suggested a supporting role for the stroma, particularly the MSCs and BM endosteal, in maintaining nascent hematopoietic cells and modulating hematopoiesis (Lord et al., 1975; Dexter et al., 1977). These early studies were supported by *in vitro*-based assays showing that the differentiated osteoblasts from BM MSCs produce cytokines necessary for supporting primitive hematopoietic cells (Taichman and Emerson, 1994).

Later investigations indicated that the BM possesses regions with specialized function or so-called “niches” that play a crucial role in regulating hematopoietic stem cells (HSCs) function. Thus far, two main BM niches, called endosteal niche and vascular niche, have been described.

## Endosteal Niche and Hematopoiesis

The endosteal niche is the inner surface of the bone surrounding BM and is composed of osteoblasts and osteoclasts, the cells that are believed to secrete factors required for hematopoietic stem cells (HSCs) function. The supportive role of the osteoblasts for HSCs has initially been studied in transgenic mouse models, and the results have shown that activation of Parathyroid hormone/Parathyroid hormone-related protein (PTH/PTHrP) receptors (PPRs) in osteoblasts increases the number of not only osteoblasts but also HSCs in a Notch-signaling dependent manner (Calvi et al., 2003). Likewise, in a separate study, conditional inactivation of bone morphogenetic protein (BMP) receptor type IA (BMPRIA) increases the number of both spindle-shaped N-cadherin^+^CD45^–^ osteoblastic cells as well as HSCs (Zhang et al., 2003). The simultaneous increase in the number of both osteoblasts and HSCs shown in these studies highlights the dependency of HSCs on osteoblasts in the endosteal niche. To explore the underlying mechanism for this dependency, several studies have investigated the factors mediating the cross-talk between osteoblasts and HSCs. For instance, the interaction of Tie-2 on the surface of HSCs with its ligand Angiopoietin-1 (Ang-1) expressed by osteoblasts has been shown to retain the HSCs in a quiescent state in the BM endosteal niche (Arai et al., 2004). The extracellular ionic calcium gradient and the calcium-sensing receptors expressed by HSCs have also been identified as critical in retaining HSCs near the endosteal niche (Adams et al., 2006).

**FIGURE 1:**
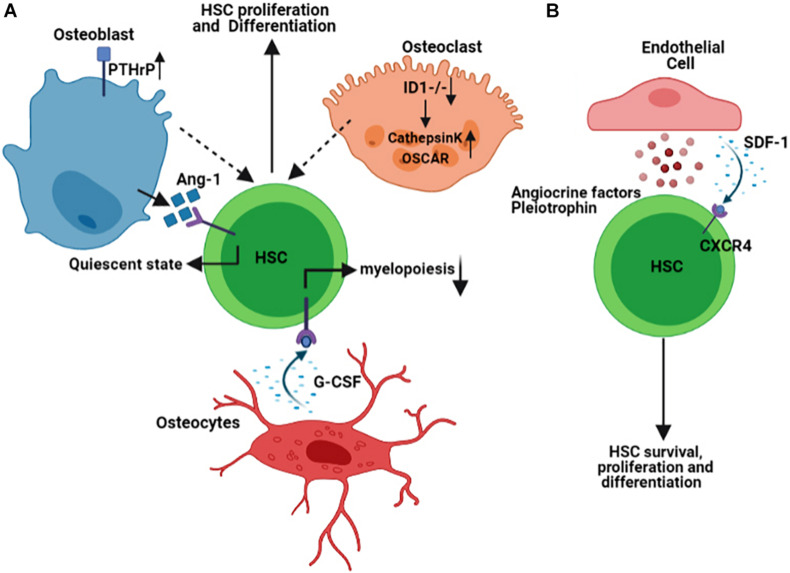
Schematic representation summarizing some of the Hematopoietic Stem Cells (HSCs) interaction with the cells of endosteal **(A)** and vascular **(B)** niches within the BM microenvironment. Figures are generated by BioRender.com.

In addition to osteoblasts, osteocytes are also an essential part of an endosteal niche. A group of researchers has identified that osteocytes restrict myelopoiesis, the expansion of myeloid lineages from the HSCs, by secreting granulocyte colony-stimulating factor (G-CSF) in a G-protein (Gsα)-mediated manner (Fulzele et al., 2013). Interestingly, bone resorbing cells or osteoclasts are also found in the endosteal niche. Remodeling of the endosteal niche by osteoclasts is not only important in normal physiology of the endosteal niche, but also important in pathophysiology of malignant cells, shown to re-activate the latent malignant cells, resistant to chemotherapy-induced cell death (Lawson et al., 2015). The modulation of hematopoiesis by osteoclasts has previously been addressed using transgenic mouse models. Mice lacking Inhibitor of differentiation protein expression (*Id1−/−*) develop osteoporotic phenotype, which is associated with upregulation of osteoclast specific genes including osteoclast-associated receptor (OSCAR), and Cathepsin K and induces HSCs proliferation and differentiation toward myeloid progenitors (Chan et al., 2009; Figure 1A). Besides, stress situations are shown to activate osteoclasts within the endosteal niche and mobilize the hematopoietic progenitors (Kollet et al., 2006). These studies indicate that remodeling of the bone, mediated either by osteoclasts and/or by osteoblasts, is tightly linked to hematopoiesis.

## Vascular Niche in the Homeostasis of Hematopoiesis

The bone marrow vasculature is composed of either arterioles, which branch from the main arterial vessels entering the marrow, or sinusoides, which are thin-walled and fenestrated vessels occupying the endosteal regions. These BM blood vessels have distinct permeability properties, capable of regulating leukocyte trafficking or maintenance of HSCs. While arterial vessels with less permeability retain the quiescent HSCs, the leaky sinusoidal vessels’ architecture allows for activation of HSCs and trafficking of leukocytes in and out of the BM (Itkin et al., 2016). Endothelial cells that line the arteriolar or sinusoidal vessels secrete several factors that maintain HSCs and modulate their function during both physiological and pathological conditions. Quiescent HSCs are closely associated with small arterioles in the BM endosteal niche. The pericytes that encapsulate these small arterioles are found to be a rare population that expresses NG2+ and are distinct from those Leptin Receptor, LepR+ pericytes found in the perisinusoidal niches (Kunisaki et al., 2013). Pericytes expressing (LepR+) are shown to produce stem cell factor (SCF, also known as KITL) that is required for maintaining HSCs in the BM (Ding et al., 2012). While their quiescence status governs the localization of HSCs in the NG2+, drug- or genetic-induced activation of HSCs distributes them from NG2+ periarteriolar to LepR+ perisinusoidal niche, underlining the significance and dynamic nature of the niche components.

The gene signature of the endothelial cells in the vascular niche is a determining factor in the lineage commitment of the HSCs. It has been demonstrated that Notch ligand delta-like ligands (*Dll1,4*) are expressed on the vascular endothelial cells of vascular niche, and deletion of *Dll4* from vascular niche primes gene expression of HSCs toward the expansion of common myeloid progenitor cells while reducing common lymphoid progenitor cells (Tikhonova et al., 2019). The angiocrine factors derived from endothelial cells are known to regulate HSCs differentiation and hematopoiesis (Rafii et al., 1995). The chemokine stromal-derived factor 1 (SDF-1) is expressed by osteoblasts in the endosteal niche and the BM’s endothelial cells. The interaction of SDF-1 with its receptor, C-X-C Motif Chemokine Receptor 4 (CXCR4), is indispensable for HSCs biology, mediating its survival, proliferation, and homing in both endosteal and vascular niches (Adams and Scadden, 2006; Teicher and Fricker, 2010). Pleiotrophin (PTN), also called Heparin-Binding Growth-Associated Molecule, is also another factor secreted by endothelial cells in the BM vascular niche and regulates both HSCs self-renewal and their homing to vascular niche (Himburg et al., 2012; Figure 1B).

The interaction between niche elements has also been a subject of interest impacting BM homeostasis and hematopoiesis. Taking advantage of conditional deletion of CXCL12 in hematopoietic or stromal cells of BM, a group of scientists has found that HSCs and early lymphoid progenitor cells occupy distinct niches in the BM (Ding and Morrison, 2013). A recent study has highlighted the complexity of the BM microenvironment at a single cell level using single-cell transcriptomic profiling and identified that the niche elements’ cellular structure is highly heterogeneous, expresses factors that regulate hematopoiesis and undergoes widespread transcriptional remodeling under stress condition (Tikhonova et al., 2019). Consistent with this observation, the stress conditions imposed by the growth of malignant cells could modify the characteristics of the BM niches, thus impacting the proper HSCs functioning.

## BM Microenvironment in Lymphoid Malignancies

The interaction between SDF-1 and CXCR4 is shown to modulate normal hematopoiesis but also mediate homing and engraftment of the tumor cells in the BM (Sipkins et al., 2005). Although the BM’s involvement with malignant B-cells differs among different lymphoid malignancies, accumulating evidence indicates that the BM microenvironment is involved in both lymphomagenesis and conferring resistance to the conventional chemo- or radio-therapeutic interventions (Mangolini and Ringshausen, 2020). BM infiltration is characterized by the formation of nodular aggregates composed of malignant B-cells mixed with CD4+ T-cells and lymphoid-like stromal cells (Vega et al., 2002). Amongst different BM components, the role of the BM mesenchymal cells in the lymphoma pathogenesis has been studied the most, and the interaction between malignant B-cells and BM stromal cells are described to be specific to the type of malignancy.

It was initially assumed that the human BM MSCs originated from a malignant clone in hematological malignancies. To test this possibility, human BM MSCs were first isolated from chronic lymphocytic leukemia (CLL) and acute lymphoblastic leukemia (ALL) patients and then characterized for the presence of potential cytogenetic abnormalities using conventional cytogenetic methods. The data, however, showed that the BM MSCs had normal karyotypes, lacking any clonal relationship with the malignant cells (Campioni et al., 2012). Likewise, another study identified that MSCs isolated from Hodgkin lymphoma or non-Hodgkin Lymphoma cells were similar to their normal counterparts in functional and phenotypic properties (Zhao et al., 2007).

Moreover, the analysis of chromosomes in MSCs isolated from multiple myeloma patients also showed no chromosomal clonal markers specific to plasma cells. However, they were shown to overproduce IL-6 and had a low capacity to suppress allogeneic T-cell proliferation capacity as compared to the cells from their normal counterparts (Arnulf et al., 2007), providing an additional line of evidence that MSCs display altered characteristics in hematological malignancies. While the chromosomal clonal abnormalities have not been identified in BM MSCs of lymphoma patients, further future characterization of these cells using state-of-the-art omics technologies could reveal the characteristics of these cells in lymphoma patients.

Recent studies have demonstrated that BM stromal cells’ response to environmental cues is compromised in lymphoma patients, and they display unique phenotypic features. For instance, the expression of CD37L (also known as 4-1BB ligand or TNFSF9) is shown to be present in the BM of the B-cell lymphomas, including mantle cell lymphoma, follicular lymphoma, B-lymphoblastic leukemia, and lymphoplasmacytic lymphoma, but not in those with reactive lymphoid aggregates, indicating that interaction of CD137/CD137L signaling may be essential in B-cell oncogenesis and could be targeted in immunotherapies (Zhao et al., 2014). Signals received by the BM mesenchymal cells can induce their protective effect toward the tumor cells through their mobilization from the BM. Isolation of BM-derived MSCs and their stimulation with tumor necrosis factor-α and lymphotoxin-α1β2 (TNFa/LT) are shown to upregulate CD54 and CD106 expression and also results in the generation of dense extracellular reticular structures that are positively stained with transglutaminase and fibronectin. Besides, treatment of these cells with TNF/LT upregulates cytokine production (Ame-Thomas et al., 2007), indicating that these cells can migrate from BM into the secondary lymphoid organs and generate lymph node-specific stromal cells in support of malignant cell survival and proliferation.

In addition to BM stromal cells, the interaction of lymphoma cells with endothelial cells in the BM vascular niche could also promote tumor cell behavior. For instance, in *the Eu-myc* + lymphoma mouse model, the secretion of fibroblast growth factor-4 (FGF4) by lymphoma cells and its binding to the cognate receptor FGFR1 on endothelial cells prompts Notch ligand Jagged1 (Jag1) expression by these cells. Jag1, in turn, induces an aggressive phenotype in the malignant lymphoma cells and confers resistance to chemo-therapeutic agents (Cao et al., 2014). This data highlights that the reciprocal interaction between tumor cells and cells of the BM niche is a critical factor in supporting the malignant phenotype. It has previously been shown that normal hematopoietic stem and progenitor cells (HSPCs) compete with acute myeloid leukemia cells for the same functional BM niche in the BM (Glait-Santar et al., 2015), however the mechanism by which the lymphoma and HSPCs compete with another awaits more investigation.

## Bone Marrow Niche in Waldenstrom Macroglobulinemia (WM)

The significance of the BM microenvironment in the pathogenesis of WM has evolved remarkably in recent years. BM is the primary location for infiltration and accumulation of the clonal immunoglobulin M (IgM)-producing lymphoplasmacytic lymphoma (LPL) cells, leading its homeostasis to be transformed toward a permissive niche for malignant cell growth and proliferation (Jalali and Ansell, 2016). The intricacy of the BM microenvironment in WM pathogenesis involves the multifaceted interactions between cells of different origins within the BM. These interactions occur directly through cell-to-cell contact or indirectly through secreted factors, such as chemokines, cytokines, and metabolites (Elsawa et al., 2011; Jalali and Ansell, 2016; Jalali et al., 2020). For instance, an excessive number of mast cells is present in the BM of WM patients, and this increase was shown to promote the WM cell expansion via the interaction between CD40 and CD40 ligand (CD154) found on the surface of malignant cells and mast cells (Tournilhac et al., 2006). Also, soluble CD27 (sCD27), a TNF family protein member elevated in the WM serum, can induce the expression of CD154 on the mast cells, further potentiating the CD40/CD152 interaction (Ho et al., 2008). In addition to mast cells, other BM immune cells, including monocytes, T-cells, natural killer cells, and dendritic cells, are also emerging to play a significant role in WM pathogenesis. Monocytes in the BM of the WM patients are shown to overexpress PD-1 ligand, PD-L1. Given that the binding of PD-1 to PD-L1 gives rise to an exhausted phenotype in T-cells, the increased PD-L1 expression by monocytes could compromise T-cell function in WM, indicating that not only the cellular cross-talk between tumor and immune cells but also the interaction between immune cells themselves contribute to WM pathogenesis.

Our group has previously studied the role of both chemokines and cytokines in WM patients and shown that the levels of IL-6, G-CSF, CCL5, and IL-21, are elevated in the tumor microenvironment of the patients, and these changes are linked to increased cell proliferation and IgM secretion by WM cells (Elsawa et al., 2011; Hodge et al., 2012). Increased expression of CCL3 or macrophage inflammatory protein-1 alpha (MIP-1α) by WM cells has also been reported on the WM BM sections, but the biological significance of this elevation awaits further evaluation. In addition to cytokines and chemokines, a very recent study from our group has shown that metabolites’ levels are also disturbed in WM patients. While a variety of different metabolites, including those involved in glucose, amino acid, and lipid metabolism are found to be changed, the differential expression and the number of the metabolites belonging to glutathione metabolism have been shown to be higher in WM than the normal subjects (Jalali et al., 2020). Elevation in glutathione metabolism protects the WM cells from high oxidative stress imposed on WM cells, thus promoting malignant cell growth (Jalali et al., 2020).

**FIGURE 2:**
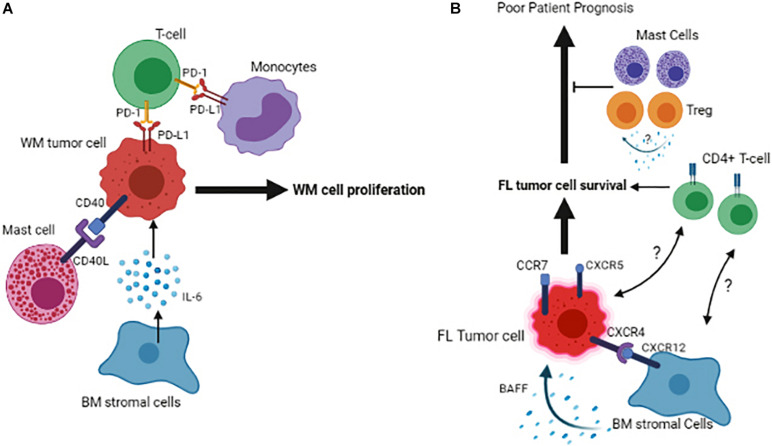
Schematic representation of the tumor cell interactions with its microenvironment in the BM of Waldenstrom Macroglobulinemia **(A)** and Follicular Lymphoma **(B)**. Figures are generated by BioRender.com.

Interestingly, we have also shown that lipids are critical players in disease progression from a pre-malignant state of Monoclonal gammopathy of undetermined significance of the immunoglobulin M class (IgM-MGUS) into the malignant state of WM (Jalali et al., 2021). In this study, most lipids and fatty acids, including those belonging to diacids, polyunsaturated fatty acids, and short-chain fatty acids, were found to be significantly reduced in the course of malignant disease transformation, and these changes were associated with altered expression of lipid metabolizing transcription factors; peroxisome proliferating factor (PPAR) family of proteins, and also lipid metabolizing enzymes such as lipoxygenases, 5-LOX, and 15-LOX. The reduced level of fatty acids and lipids was associated with increased lipid peroxidation, providing additional evidence that the tumor microenvironment in WM is under oxidative stress (Jalali et al., 2021). These accumulating data highlight the significance of microenvironmental elements in malignant cell growth and WM disease progression (Figure 2A).

## Marginal Zone Lymphoma (MZL) and BM Microenvironment

Marginal zone lymphoma (MZL) is a malignancy of mature B-cells and has an indolent clinical presentation. Like WM, the malignant clone can localize in the BM microenvironment, where they find a permissive niche for survival and resistance to therapy. Therefore, the interaction of tumor cells and BM stromal cells could be critical in MZL tumor biology. To date, minimal information is available to describe the underlying molecular mechanisms of MZL cell localization and their subsequent survival/proliferation in the BM. It has been shown that BM stromal cells express CD40 and recruit CD40L-expressing mast cells into the BM aggregates containing MZL cells. This interaction is shown to support the mature B-cell proliferation and results in increased inflammatory response and unfavorable patient outcome (Franco et al., 2014). Comparing the BM MSCs isolated from MZL patients with those of healthy individuals has shown that they do not differ in their differentiation capacity and phenotypic features; however, their growth capacity is compromised due to increased apoptosis. This implies that tumor cells may disseminate soluble factors that could specifically impact mesenchymal stromal cell proliferation in the BM. On the other hand, BM mesenchs also increase the proliferation of malignant B-cells, and this effect is believed to be potentially mediated by the secretion of the soluble factors from MSCs. The cytokines CXCL-12 and VACM-1 that mediate the homing of malignant B-cells in the BM do not show any differential expression between BM MSCs obtained from patients and healthy donors, indicating that they are possibly less important in chemoattraction of the MZL cells into the BM (Pontikoglou et al., 2019). Further studies are necessary to identify the differentiating molecules that recruit the tumor cells into the BM and specific phenotypic markers of both tumor and BM microenvironment cells that could potentially be targeted in MZL.

## BM Niche in Follicular Lymphoma (FL)

Follicular lymphoma (FL) is an indolent B-cell lymphoma arising from the malignant transformation of germinal center (GC) B-cells and involves lymph nodes and BM, resulting in approximately 70% of follicular lymphoma patients showing BM infiltration at diagnosis. Based on genetic analysis, over 90% of the FL cases exhibit an error in V(D)J recombination in the BM, which results in a BCL2/IGH translocation (Roulland et al., 2011), however, this translocation on its own is not sufficient for malignant transformation and additional factors such as, but not limited to, microenvironmental elements may play a role in the development of this disease. Likewise, FL cells fail to grow *in vitro* due to the lack of extrinsic stimuli, highlighting the role of the microenvironment cells and soluble factors in promoting cell survival and proliferation. Initially, microarray analyses demonstrated that molecular characteristics of the microenvironment cells are strong predictors of clinical outcome in FL patients (Dave et al., 2004). This observation has been extended by recent data indicating that the phenotypic signature of non-malignant microenvironment cells is associated with altered anti-tumor response. In this respect, expression of LAG3 and TIGIT by T-cells is shown to define a response to immunotherapy and determine patient outcome in FL (Yang et al., 2017, 2020). However, the significance of the BM in this context remains to be addressed. In an attempt to show the relevance of immune cells within the BM, a group of investigators has shown that a diminished number of macrophages and cytotoxic T-cells in the lymph nodes promote the propensity of the tumor cells to infiltrate BM (Rajnai et al., 2012). The presence of malignant B-cells in the BM in FL patients is shown to be associated with an increased CD4+/CD8+ T-cell ratio, indicating that an enriched CD4+ niche may favor FL cell survival (Wahlin et al., 2012).

Furthermore, pre-treatment BM infiltrates of FL patients also contain regulatory T-cells (Treg) and mast cells (Laurent et al., 2007). Given that Tregs’ presence in the tumor microenvironment of FL patients is associated with increased overall survival (Carreras et al., 2006), the enrichment of these cells in the BM may indicate a favorable response to therapy. However, the molecular mechanism of this effect remains unexplored (Laurent et al., 2007; Figure 2B).

It has been shown that the bidirectional malignant B-cell trafficking between lymph node and BM leads to cancer relapse in FL (Wartenberg et al., 2013). Data show that the FL cells in the BM have a low proliferation index compared to the ones residing in the lymph nodes. The B-cells’ subclones that occupy the lymph node are different from those in the BM, indicating that the BM microenvironment provides a niche for selecting specific clonal tumor cells in FL (Bognar et al., 2005). One of the reasons for this selection could be that distinct FL clones find BM niches as a milieu for their quiescence and therefore remain protected from induced cell death by therapeutic agents. However, it remains to be answered whether the immune or stromal cells of the BM are involved in promoting this niche. FL tumor cells expressing CXCR4, CXCR5 and CCR7 home in both BM and LN, and the interaction of CXCR12 expressed on BM stromal cells of the lymph node/BM with CXCR4 expressed by tumor cells, has been found to play a critical role in this context (Ame-Thomas et al., 2007). Once the BM is infiltrated, malignant lymphoid aggregates containing ectopic LN-like reticular cells are developed (Vega et al., 2002). Consistent with this, the MSCs isolated from the BM of FL patients display a fibroblastic reticular cell phenotype and can support the growth of malignant B-cells more efficiently than the ones isolated from healthy individuals. One of the underlying mechanisms of this support could be the factors that are secreted by BM stromal cells and mediate the survival of malignant B-cells in FL, as it has been shown that adhesion of lymphoma cells to BM stromal cells triggers the secretion of B cell-activating factor (BAFF) by BM stromal cells and BAFF, in turn, acts as a survival factor for malignant B-cells (Lwin et al., 2009).

## Diffuse Large B-Cell Lymphoma (DLBCL) and BM Microenvironment

Diffuse Large B-cell Lymphoma (DLBCL) is an aggressive and the most prevalent type of non-Hodgkin lymphoma (NHL) comprising 30–40% of all cases and genetically represents a heterogeneous group of tumors. Initial gene expression profiling has identified two distinct subtypes of DLBCL, including the germinal center B cell-like (GCB) and activated B cell-like (ABC) (Alizadeh et al., 2000). Approximately 30% of FL transform into aggressive diffuse large B cell lymphomas (DLBCL), in which the malignant cells are less dependent on their microenvironment. Though being a rare event, the infiltration of the BM with malignant B-cells also occurs in DLBCL, implying that tumor cells may find BM as an advantageous location for their survival. It has been shown that there are anergic B-cells in the BM of the patients with DLBCL that are CD21^–^/low/CD38^–^ and their increased number is associated with shorter overall survival. Interestingly this number is lower in GCB-DLBCL than ABC-DLBLC. Given the poorer survival prognosis in ABC-DLBCL, this reduction could explain one of the underlying mechanisms influencing patient survival (Rijal et al., 2020).

**FIGURE 3:**
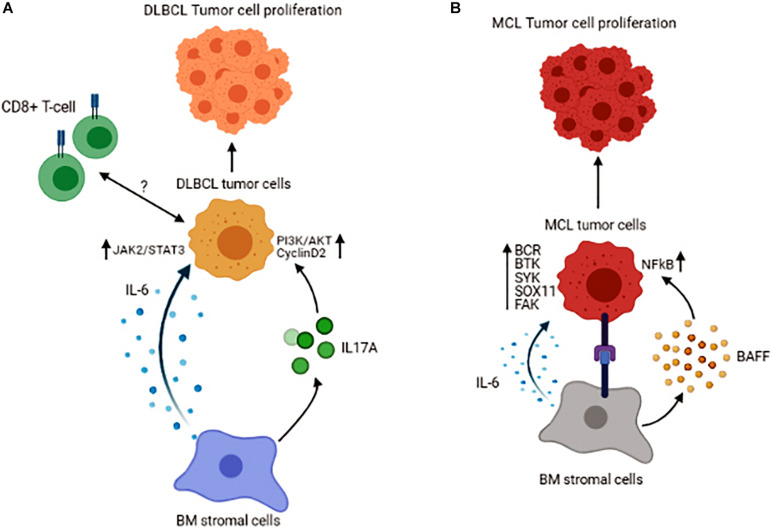
Schematic representation of the tumor cell interactions with its microenvironment in the BM of Diffuse Large B-cell Lymphoma **(A)** and Mantle Cell Lymphoma **(B)**. Figures are generated by BioRender.com.

Tumor cells could recruit other immune cells into the BM, as an increased number of the immune cells, including CD3+ T-cells, CD8+ T-cells, and CD168+ macrophages, have been found in DLBCL patients with BM involvement. In these patients, CD8+ T-cells were associated with inferior overall survival, indicating that BM infiltration of CD8+ T-cells could serve as a prognostic marker for patients with DLBCL (Jeong et al., 2017). The role of the BM stromal cells is also prominent in DLBCL. A recent study reported that co-culture of DLBCL cells with stromal cells leads to highly tumorigenic cells (Lin et al., 2018). The mechanism by which the BM stromal cells could induce DLBCL cell growth may be mediated by secreting IL-6 and in a JAK2/STAT3 dependent manner shown in both *in vivo* and *in vitro* systems. Also, BM stromal cells increase IL-17A levels, and this cytokine upregulates DLBCL cell growth through activation of cyclin D2 and PI3K/Akt signaling. In this study, concomitant stimulation of DLBCL cells with IL-6 and IL-17A was shown to exert a synergistic effect on promoting the growth and increasing cell drug-resistance *in vitro* (Zhong et al., 2019). Like FL, the interaction of lymphoma cells with BM BAFF sustains tumor cell survival in DLBCL (Figure 3A). As a result, inhibition of BAFF secretion or its neutralization is shown to sensitize the malignant cells to drug-induced apoptosis (Lwin et al., 2009). Interestingly, in DLBCL patients without BM involvement, the properties of MSCs are also changed, indicating that humoral factors in the circulation of the DLBCL patients could alter the features of MSCs (Fastova et al., 2019).

## BM Microenvironment in Mantle Cell Lymphoma (MCL)

Mantle cell lymphoma (MCL) comprises 6–8% of all non-Hodgkin lymphomas and is characterized by translocation t(11;14)(q13;q32), which results in cyclin D1 overexpression (Burger and Ford, 2011). Patients with MCL have a distinct clinical presentation, with almost 90% of them manifesting BM involvement at diagnosis, again underlining the fact that the BM provides a supportive niche for their growth and proliferation. The interaction between malignant B-cells and BM MSCs in MCL has been shown to promote tumor cell survival and give rise to resistance to chemo-therapeutic agents, effects that are mediated by BAFF and activation of NFκB signaling pathway (Medina et al., 2012). In a series of other studies, B-cell receptor (BCR)-mediated signaling together with constitutive activation of Bruton’s tyrosine kinase (BTK) and Spleen Associated Tyrosine Kinase (SYK) are linked to cell proliferation and malignant cell adhesion to the BM stromal cells in MCL (Rinaldi et al., 2006; Bernard et al., 2015). These data highlight the fact that B-cell signaling is an essential part of the interaction between BM stromal and MCL cells. Based on recent molecular studies, a novel molecule known as SOX11 [SRY (sex determining region-Y)-box11] is identified as an oncogene involved in the pathobiology of MCL. It has been demonstrated that SOX11 stimulates the interaction between MCL cells and stromal cells and facilitates homing of the malignant cells to the BM in a CXCR4 and FAK signaling-dependent manner (Balsas et al., 2017). The significance of SOX11 in MCL pathogenesis has also been explored using a transgenic mice model expressing SOX11 under a B-cell-specific promoter. These mice are shown to develop a B-cell hyperplastic phenotype that involves the BM, spleen, and peripheral blood and resembles human MCL in that they exhibit a CD5+/CD19+/CD23− phenotype and display activated BCR signaling (Kuo et al., 2018). Increased expression of SOX11 is correlated with inferior prognosis in patients with MCL. Furthermore, cytokines in the tumor microenvironment also contribute to the survival of MCL cells. It has been reported that IL-6, in both autocrine and paracrine fashion, improves the survival and proliferation of the lymphoma cells (Zhang et al., 2012; Figure 3B).

Another target molecule that mediates incoming signals from the BM microenvironment to the malignant cells is focal adhesion kinase (FAK). In MCL, increased FAK expression by lymphoma cells correlates with increased incoming signals from BM stromal cells. These signaling cascades, which involve several kinases, including AKT, MAPK p42/44, and NFκB, result in an increased survival and proliferation capacity of the malignant B-cells and confers resistance to therapeutic agents (Rudelius et al., 2018). Moreover, *in vitro* based studies have shown that a Hedgehog (Hh) inhibitor, LDE225, suppresses both migration and retention of the MCL cells in the BM, and this effect is shown to be mediated by inhibiting FAK signaling in MCL cells and suppressing IL-6, SDF-1, and VCAM-1 expression by BM stromal cells. However, the inhibition of BM involvement by the Hedgehog inhibitor is not associated with reduced proliferation of MCL cells, instead it is due to increased = −094321 autophagy that increases the cell survival (Zhang et al., 2016). Even though these studies are performed in different settings, the common signaling molecules, including SDF-1, IL-6, and FAK, appear to be central in malignant cell growth in MCL patients’ BM.

## Clinical Relevance

HSPCs are clinically being transplanted into the patients to restore hematopoiesis and an understanding of the BM environment has been leveraged to improve this process. Autologous hematopoietic stem cell transplantation (HSCT) is a well-known strategy to treat patients with hematological malignancies, including lymphomas, and is achieved by mobilizing and collecting stem cells prior to intensive chemotherapy. Mobilization is generally achieved using cytokines, granulocyte-colony stimulating factor (G-CSF) or granulocyte macrophage-colony stimulating factor (GM-CSF), alone or in combination with plerixafor to dislodge HSPCs from the BM niches (Giralt et al., 2014). The underlying mechanism of action of these cytokines and plerixafor is by disrupting the interaction of CXCR4 and SDF-1, resulting in HSPCs release into the systemic circulation (Petit et al., 2002; Steinberg and Silva, 2010). However, mobilization of the HSPCs from the BM niche is a complex phenomenon and could be influenced by several factors rather than mediated by SDF-1/CXCR4 interaction. For instance, a polymorphism of the CD44 gene is shown to impact CD34+ HSPCs cells mobilization from the BM niche into systemic circulation in hematological malignancies, with some alleles having poorer CD34+ cell mobilization than others (Szmigielska-Kaplon et al., 2014). To induce the anti-tumor reactive T-cells, T-cell therapies hold great promise in clinical applications. However, one of the critical aspects of successful T-cell transplantation into the patient is that they persist (longevity), differentiate, and remain functionally active in the recipient organism. A recent study shows that overexpression of CXCR4 in CD8+ T-cells redirects them to CXCR12+ cells in the BM vascular niche and promotes their memory differentiation and anti-tumor response (Khan et al., 2018). Instead, use of CXCR4-targeting agents holds promise for treatment of the patients with B-cell lymphomas including WM (Castillo and Treon, 2020). Chemotherapeutic agent, such as ibrutinib that targets B-cell receptor signaling and is used in the treatment of certain B-cell lymphomas could also interfere with the interaction of tumor cells with the BM niche. This notion is supported by a study, showing that the downregulation of B-cell receptor signaling is associated with a reduced expression of CD44, which a marker for homing of the cells in the BM niche, in B-cell acute lymphoblastic leukemia (Kim et al., 2017). Further studies are warranted to evaluate the balance of contradictory effects of CXCR4 inhibitors on the function of T-cell and malignant cell and also on the patient outcome.

## Conclusion

This review summarized the available data as to how the BM microenvironment could favor the disease pathogenesis predominantly in malignant lymphomas. As discussed, multiple signaling molecules and pathways promote BM localization and subsequent tumor cell growth and proliferation within the BM niche in FL, MCL, WM, MZL, and DLBCL. While these studies are mainly focused on the interaction of the BM stromal cells with malignant tumor cells, the information surrounding different microenvironment components, including immune cells of different origins or the role of soluble factors, is not fully explored in these cancers. Furthermore, the presence of genetic alterations specific to each of these malignancies likely generates a bidirectional relationship with the tumor cells resulting in a microenvironment that is unique to each particular type of lymphoma. Identifying the key and convergent molecules that engage all the microenvironment components for the advantage of the tumor cell survival is a critical step in designing the therapeutic strategies specific to each lymphoma. Such future research may identify novel targets to disrupt BM niches that promote malignant cell growth and may result in new therapies that improve the outcome of patients with lymphoid malignancies.

## Author Contributions

Both authors listed have made a substantial, direct and intellectual contribution to the work, and approved it for publication.

## Conflict of Interest

The authors declare that the research was conducted in the absence of any commercial or financial relationships that could be construed as a potential conflict of interest.

## Publisher’s Note

All claims expressed in this article are solely those of the authors and do not necessarily represent those of their affiliated organizations, or those of the publisher, the editors and the reviewers. Any product that may be evaluated in this article, or claim that may be made by its manufacturer, is not guaranteed or endorsed by the publisher.
